# Effects of Environmental Temperature Variation on the Spatio-Temporal Shoaling Behaviour of Adult Zebrafish (*Danio rerio*): A Two- and Three-Dimensional Analysis

**DOI:** 10.3390/ani15142006

**Published:** 2025-07-08

**Authors:** Mattia Toni, Flavia Frabetti, Gabriella Tedeschi, Enrico Alleva

**Affiliations:** 1 Department of Biology and Biotechnologies “Charles Darwin”, Sapienza University, 00185 Rome, Italy; 2 Department of Medical and Surgical Sciences—DIMEC, University of Bologna, 40126 Bologna, Italy; flavia.frabetti@unibo.it; 3 Department of Veterinary Medicine and Animal Science (DIVAS), Università degli Studi di Milano, Via dell’Università 6, 26900 Lodi, Italy; gabriella.tedeschi@unimi.it; 4 CRC “Innovation for Well-Being and Environment” (I-WE), Università degli Studi di Milano, 20126 Milan, Italy; 5 Centre for Behavioural Sciences and Mental Health, Istituto Superiore di Sanità, Viale Regina Elena 299, 00161 Rome, Italy; enrico.alleva@guest.iss.it

**Keywords:** zebrafish, *Danio rerio*, behaviour, temperature, environment

## Abstract

Global warming is driving significant changes in aquatic ecosystems, where temperature fluctuations influence biological processes across multiple levels of organisation. As ectothermic organisms, fish are particularly susceptible, with even minor thermal shifts affecting their metabolism, behaviour, and overall fitness. Understanding these responses is essential for evaluating the ecological and evolutionary consequences of climate change. This study investigates the effects of acute (4-day) and chronic (21-day) exposure to three temperature regimes—18 °C (low), 26 °C (control), and 34 °C (high)—on the spatio-temporal shoaling behaviour of adult zebrafish (*Danio rerio*). Groups of four fish were tested for six minutes in water maintained at the same temperature as their prior acclimation. Shoaling behaviour was assessed by analysing shoal structure—encompassing shoal dimensions and cohesion—as well as spatial positioning. Parameters measured included inter-fish distance, shoal volume, shoal area, homogeneity index, distance to the centroid, and the shoal’s vertical and horizontal distribution. Results revealed complex behavioural changes influenced by both temperature and duration of exposure. At 18 °C, zebrafish showed a marked preference for the bottom zone and exhibited no significant temporal modulation in exploratory behaviour—patterns indicative of heightened anxiety-like responses. In contrast, exposure to 34 °C resulted in increased shoal cohesion, particularly under chronic conditions, and a progressive increase in environmental exploration over the six-minute test period. This enhancement in exploratory activity was especially evident when compared to the first minute of the test and was characterised by greater vertical movement—reflected in the increased use of the upper zone—and broader horizontal exploration, including more frequent occupation of peripheral areas. These findings align with previous research linking thermal variation to neurobiological and proteomic alterations in zebrafish. By elucidating how temperature modulates social behaviour in ectotherms, this study offers valuable insights into the potential behavioural impacts of climate change on aquatic ecosystems.

## Simple Summary

Rising global temperatures are affecting aquatic life, especially fish, whose body temperature depends on the environment. This study looked at how adult zebrafish behave in groups (shoals) after being kept in water at different temperatures—cool (18 °C), normal (26 °C), or warm (34 °C)—for either a short time (4 days) or a long time (21 days). The fish were then observed for six minutes to see how they moved and stayed together. At the cool temperature, fish stayed near the bottom of the tank and explored less, suggesting they were more anxious. In contrast, fish exposed to warm temperature showed enhanced shoal cohesion. These patterns show that temperature affects how fish behave in groups and how they explore their surroundings. Understanding these changes is important because fish behaviour plays a key role in survival, feeding, and reproduction. This research helps us predict how climate change may impact fish and other cold-blooded animals in lakes, rivers, and oceans.

## 1. Introduction

Global warming is driving significant shifts in Earth’s climate, with aquatic ecosystems particularly vulnerable to temperature fluctuations. These variations arise from both natural environmental changes and human-induced climate change, affecting biological organisation at multiple levels. Ectothermic animals, such as fish, are especially susceptible since their physiological processes are directly influenced by external temperatures. Even minor thermal changes can impact cellular functions, metabolism, behaviour, and overall fitness, ultimately influencing population dynamics and species distributions. Understanding how ectotherms respond to temperature variations is therefore essential for evaluating the ecological and evolutionary consequences of climate change.

Studying the effects of temperature on biological processes requires controlled experimental conditions that allow for the precise regulation of variables and the reproducibility of findings. Laboratory research provides a crucial framework for understanding thermal adaptation, physiological tolerance, and the mechanisms underlying species’ responses to environmental stressors.

Among teleosts, certain species serve as key models in biomedical and ecological research due to their physiological diversity, ease of maintenance, and genetic accessibility. The zebrafish (*Danio rerio*), a small freshwater cyprinid, is widely used in research for its genetic homology with humans, rapid development, and transparent embryos, which facilitate in vivo studies [1]. This species currently serves as a common model in vertebrate biology. However, beyond its biomedical relevance, the zebrafish is an excellent model for investigating the effects of thermal variations on both physiological and ecological scales.

Zebrafish exhibit broad thermal tolerance, with viability ranging from 6 °C to 43 °C and survival at temperatures between 4.5 °C and 42 °C [2,3,4]. This wide thermal range allows researchers to test variations within the species’ vital limits, providing insights not only into physiological responses but also into ecologically relevant scenarios. In their native habitats in South Asia [5], zebrafish experience daily temperature fluctuations of up to 5 °C and seasonal variations from 6 °C in winter to over 38 °C in summer [6]. They inhabit diverse freshwater environments, including brooks, lakes, ponds, and rice fields [7,8], where water temperatures typically range from 16.5 °C to 34.0 °C [9]. These natural conditions make zebrafish an ideal species for studying physiological and behavioural responses to ecologically relevant temperature changes [10].

As ectotherms, zebrafish rely on environmental temperatures to regulate their metabolic and physiological functions. Their exposure to natural thermal fluctuations provides a unique opportunity to examine molecular, physiological, and behavioural responses to temperature shifts. Research on zebrafish thus contributes to a broader understanding of thermal adaptation, with direct implications for assessing the impact of global warming on aquatic ectotherms.

Zebrafish are valuable not only for biochemical and physiological studies but also for behavioural research, particularly in investigating the effects of environmental temperature changes. They are increasingly used in behavioural studies due to the development of validated assays comparable to those in rodent models. Tests such as the novel tank diving test, the light–dark preference test, the mirror biting test, and the Y-maze test have been employed to assess anxiety-like behaviour, exploratory activity, aggression, and cognitive function [11,12,13,14].

Social behaviour is a key aspect of zebrafish ecology, as they naturally form shoals [15,16,17]. In the wild, shoal sizes vary depending on environmental factors, ranging from small groups of 4–12 individuals to large aggregations of ~300 fish [7,18]. Shoaling confers multiple survival advantages, including improved predator avoidance, enhanced foraging efficiency, and increased reproductive success [19,20,21]. Moreover, shoaling enhances group coordination in response to threats, facilitating collective escape behaviours [22,23,24].

Several behavioural assays have been developed to assess zebrafish sociality, including the social preference test, which measures an individual’s attraction to conspecifics, and the shoaling test, which evaluates group cohesion. The shoaling test is particularly relevant for detecting environmental stressors that influence social organisation. Various factors, such as food availability, predator presence [17], and alarm substances [25], have been shown to affect shoaling behaviour, making it a valuable indicator of stress. Shoaling dynamics are also influenced by changes in biotic and abiotic factors, including temperature fluctuations [17,18,26,27,28,29]. Investigating these aspects enhances our understanding of zebrafish social behaviour and reinforces their role as a behavioural model.

The aim of this study is to assess the effects of acute (4 days) and chronic (21 days) exposure to three different temperature conditions—18 °C (low-temperature treatment), 26 °C (control), and 34 °C (high-temperature treatment)—on the shoaling behaviour of adult zebrafish. These temperatures were selected based on the work of Vergauwen et al. [30], as they fall within the zebrafish’s vital thermal range and reflect ecologically relevant temperatures encountered in their natural environment. The control temperature was set at 26 °C, as this corresponds to the species’ thermal preference [3].

This study builds upon previous research demonstrating that exposure to the same thermal conditions (18 °C vs. 26 °C and 34 °C vs. 26 °C) induces significant alterations in the zebrafish brain proteome and lipidome, alongside behavioural changes indicative of neurotoxic effects associated with temperature variations [13,14,31,32,33,34].

Unlike previous studies that focused on the behavioural responses of individual zebrafish [13,14], the present research investigates how thermal variation affects the dynamics of small shoals, each composed of four individuals. By employing a three-dimensional behavioural analysis, this study adopts a group-level perspective that not only offers greater ecological validity but also reveals new insights into the collective responses to thermal stress.

In contrast to earlier work by Angiulli et al. [13] and Nonnis et al. [14], which relied on individual-based assays, our approach allows the characterisation of social interactions under temperature challenge, thereby extending the understanding of zebrafish behaviour within a more naturalistic and ethologically relevant framework.

These findings, considered alongside existing evidence on temperature-related neurophysiological changes in zebrafish, contribute to a broader understanding of how thermal stress modulates both neural function and social behaviour in ectothermic species. This, in turn, has important implications for interpreting the potential effects of climate-driven environmental changes on aquatic animal communities.

## 2. Materials and Methods

### 2.1. Subjects and Housing Conditions

A total of 72 adult (6–7-month-old) wild-type zebrafish from a stock colony purchased from commercial dealers were used in the present study. The sex ratio was approximately 50:50 (male:female), and the mean weight was 0.4 g. Fish were randomly assigned to three tanks (40 cm × 30 cm × 30 cm, width × depth × height) with a capacity of 33 L (home tanks) and maintained at 26 ± 1 °C (control temperature) for 10 days to acclimate to the tanks (adaptation period). Each tank housed 24 fish, which were kept under an artificial photoperiod (14:10 light/dark cycle) and fed three times a day (10:00, 14:00, and 18:00) with commercial dry granular food (TropiGranMIX, DAJANA PET, s. r. o., Bohunovice, Czech Republic) using automatic fish feeders (Eden 90, Eden Water Paradise, Vicenza, Italy), allowing the fish to feed ad libitum until satiation.

The water used throughout the experimental phase was produced by reverse osmosis pumps (Reverse Osmosis AquiliOS2, Ancona, Italy) and adjusted to the appropriate salinity by adding aquarium salt (1 g/l, Aqua Medic 301.01, Bissendorf, Germany). To ensure optimal water quality, a constant flow of filtered water (600 L/h) was maintained in each tank using external filter systems (Eden 511 h, Eden s.r.l., Vicenza, Italy), and water was continuously aerated (7.20 mg O_2_/L) using an aquarium aerator (Sicce AIRlight, SICCE s.r.l, Vicenza, Italy, 3300 cc/min 200 L/h). The chemical and physical characteristics of the tank water were monitored at least twice per week, measuring parameters such as water hardness, pH, ammonium (NH_4_), ammonia (NH_3_), nitrate (NO_3_), nitrite (NO_2_), phosphate (PO_4_), copper (Cu), and calcium (Ca^2+^) using the Sera Aqua-Test Box Kit (Sera GmbH, Heinsberg, Germany) and an eSHa Aqua Quick Test (eSHa Lab, Maastricht, The Netherlands). Faeces and uneaten food were removed from the tanks at least three times per week. During tank-cleaning operations, a water exchange of approximately 20–30% per week was performed to maintain water volume and ensure stable chemical–physical parameters.

### 2.2. Thermal Treatment

The three home tanks were randomly assigned to three different temperature conditions: 18 °C, 26 °C, and 34 °C. The water temperature was gradually adjusted from 26 °C to 18 °C or 34 °C over a period of 72 h. Fish were then maintained at the respective experimental temperatures (18 ± 1 °C and 34 ± 1 °C) for 21 days, while fish kept at 26 ± 1 °C served as controls. The three temperature conditions were selected based on Vergauwen et al. [30], as they fall within the zebrafish’s vital range and correspond to temperatures encountered in their natural environment. Water temperature was regulated using digital thermostats (Eden 430, Eden s.r.l., Vicenza, Italy) connected to heating coils (Eden 415, 230 V, 50/60 Hz, 80 W, Eden s.r.l., Vicenza, Italy) supplemented by a cooling system (TK 150, TECO S.r.l., Ravenna, Italy). Additionally, water temperature was checked daily with a hand thermometer. The internal structure of each tank (comprising heating coils, filter inlet and outlet pipes, and aerators) was standardised across all tanks to ensure uniform environmental conditions. No fish mortalities occurred during the thermal treatments. All experimental procedures were approved by the Animal Care Committee and authorised by the Italian Ministry of Health (protocol number 290/2017 PR).

### 2.3. Shoaling Behaviour Task

The shoaling behaviour task was conducted following the protocol previously used by [35]. Briefly, the experimental tank (20 cm × 15 cm × 20 cm, width × depth × height) was filled with water to a depth of 10 cm, maintained at the same experimental temperature as the home tanks (18 °C, 26 °C, or 34 °C, depending on the experimental group). Preliminary tests confirmed that water temperature remained stable throughout the 6 min test duration.

On the fourth and twenty-first day from the beginning of the thermal treatment, groups of four zebrafish were transferred to the experimental tank to undergo the shoaling behaviour test. All testing was performed between 10:00 and 14:00 h each day. Fish were gently netted from their home tank and transferred in a plastic beaker to the testing tank. They were then gently poured into the centre of the tank. Each trial lasted 6 min and was recorded using two orthogonally positioned webcams (Logitech C922, Logitech International S.A., Lausanne, Switzerland): one placed 60 cm above the tank (top view) and the other 60 cm from the back wall of the tank (frontal view). Both cameras were connected to separate laptops, and recordings were initiated simultaneously to ensure synchronised video footage from both perspectives.

At the end of each test, the four experimental subjects were transferred to a waiting tank with water conditions identical to those of the home tank, including temperature. After each trial, the experimental tank was emptied, rinsed, and refilled with clean water at the appropriate experimental temperature. At the end of the behavioural test, when all 24 fish had been analysed, the fish were returned to the home tank.

It is important to note that, for each temperature group, all 24 fish were assessed both after 4 days (acute exposure) and after 21 days (chronic exposure) from the onset of thermal treatment. However, the composition of the shoals—i.e., the four individuals comprising each group—differed between the acute and chronic assessments. As a result, direct comparisons between acute and chronic data were not conducted, primarily due to the variation in shoal composition and the fact that the fish tested during the chronic phase had already been exposed to the testing tank during the acute phase. This prior experience may have influenced their behaviour independently of the thermal treatment.

In particular, the testing tank represented a novel environment for acutely exposed fish, whereas it was already familiar to those assessed during the chronic phase. Such differences in environmental familiarity are known to affect anxiety-related responses and exploratory behaviour, potentially confounding the interpretation of temperature effects. For these reasons, data from the acute and chronic phases were analysed separately, and no direct statistical comparisons were made between them. This approach ensures that behavioural differences are interpreted within the specific context of each exposure period, thereby minimising the influence of prior experience and variation in shoal composition.

### 2.4. Three- and Two-Dimensional Analysis of Shoaling Behaviour

The recorded videos were subsequently processed by extracting screenshots every 10 s throughout the trial (resulting in 36 pairs of top-view and frontal-view screenshots per shoal).

The analysis of screenshots from synchronised frontal and top-view recordings was conducted following the protocol described in [35]. Images were analysed using ImageJ 1.53e software for Windows™ to determine the spatial coordinates of individual fish: “x and y” for the frontal camera and “x and z” for the top camera. The software was calibrated using the known dimensions of the apparatus.

For frontal view images, the tank was divided into three equal horizontal zones (bottom, middle, and top) to assess the vertical distribution of fish. For top-view images, the tank was divided into three horizontal zones, the periphery (less than 2.5 cm from the edge of the tank), the intermediate area (between 2.5 cm and 5 cm from the edge), and the centre (more than 5 cm from the edge), to measure the horizontal distribution of fish.

The individual fish coordinates were used to calculate inter-fish distance in both three-dimensional (3D) and two-dimensional (2D) perspectives, shoal volume (3D), and shoal area in both frontal and top views (2D). Calculations were performed based on the work of Rosa and collaborators [35] and using the Excel file published by the same authors [35].

### 2.5. Statistical Analysis

Shoaling behavioural parameters were expressed as means ± standard error of the mean (S.E.M.). Statistical analyses were conducted separately for the acute and chronic exposure conditions, using either one-way ANOVA or one-way repeated measures ANOVA, as appropriate.

To evaluate the effect of temperature on shoaling behaviour, the values recorded every 10 s during the entire 6 min trial (36 data points per trial) were averaged and subjected to one-way ANOVA followed by Tukey’s post hoc test for multiple comparisons.

The effect of time was assessed via temporal analysis based on the area under the curve (AUC) of the 10 s inteval data. AUC values were summed for each minute of the trial, and the resulting values were analysed by one-way repeated measures ANOVA within each temperature group. When significant effects were detected, Bonferroni’s post hoc test was applied to identify specific time points showing significant differences.

Post hoc power analyses were performed using G*Power (ver. 3.1) [36] to determine the sensitivity of the statistical tests based on the sample size and observed effect sizes.

All results are presented in the figures as means ± S.E.M. Statistical significance was set at *p* ≤ 0.05 for all tests. The summary of statistical results (F, *p*, and statistical power [1 − β]) for the behavioural parameters is reported in Table S1.

Statistical analyses were performed using OriginPro 2018 (version 95E; OriginLab Corporation, Northampton, MA, USA), RStudio (version 2023.12.1+402; RStudio Team, Boston, MA, USA) and G*Power (ver. 3.1). Figures were created using Microsoft PowerPoint 365 (Microsoft Corporation, Redmond, WA, USA).

## 3. Results

Spatio-temporal shoaling behaviour in adult zebrafish (*Danio rerio*) was analysed following exposure to three different temperature conditions (18 °C, 26 °C, and 34 °C), administered under either acute (4-day) or chronic (21-day) regimes. The analysis was structured around two main components: shoal structure and shoal positioning.

Shoal structure was assessed by evaluating both shoal dimension and shoal cohesion. Shoal dimension was measured using three parameters: inter-fish distance, assessed in three-dimensional space as well as in the two-dimensional vertical and horizontal planes; shoal volume; and shoal area, calculated in both vertical and horizontal planes. Shoal cohesion was evaluated using two metrics, distance to the centroid and the homogeneity index, both of which provide insight into the internal consistency and compactness of the group.

Shoal positioning was determined by analysing the spatial distribution of the shoal across both the vertical and horizontal planes of the tank.

To investigate whether thermal exposure significantly affected shoaling behaviour, a one-way ANOVA was performed separately for acute and chronic conditions, with temperature as the independent factor. Where significant effects were found, Tukey’s post hoc test was applied to identify specific differences between temperature groups.

In addition, a temporal analysis was conducted using one-way repeated-measures ANOVA to evaluate whether the time factor (i.e., minute-by-minute progression throughout the six-minute test) significantly influenced the measured parameters. To better capture overall behavioural trends during the trial, these analyses were based on the area under the curve (AUC) values. Where relevant, Bonferroni’s post hoc test was used to pinpoint the specific time points at which significant changes occurred.

This intra-temperature approach allowed for a detailed examination of how shoal structure and positioning evolved over time within each thermal condition, offering a comprehensive understanding of the temporal dynamics that govern zebrafish shoaling behaviour in response to temperature variation.

### 3.1. Shoal Structure

#### 3.1.1. Inter-Fish Distance Analysis

**Figure 1:**
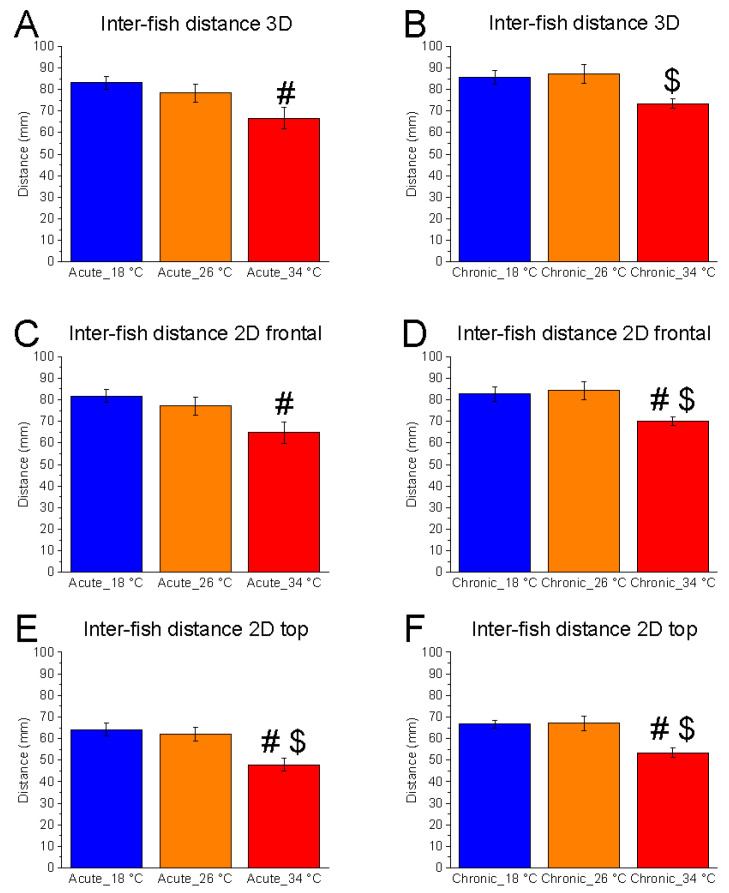
Effects of temperature on inter-fish distance. Effects of acute (**A**,**C**,**E**) and chronic (**B**,**D**,**F**) thermal exposure on inter-fish distance in 3D space (**A**,**B**), 2D vertical plane (**C**,**D**), and 2D horizontal plane (**E**,**F**). Detailed statistical values are reported in Table S1. Statistical significance: *p* ≤ 0.05. * 18 °C vs. 26 °C; # 18 °C vs. 34 °C; $ 26 °C vs. 34 °C. Colours indicate experimental temperatures: blue = 18 °C, orange = 26 °C, red = 34 °C.

A significant effect of temperature variation was observed under both acute and chronic conditions across all spatial dimensions. In three-dimensional space, inter-fish distance was significantly affected under acute (F_(2, 15)_ = 4.03, *p* = 0.0398, power (1 − β) = 0.710) and chronic (F_(2, 15)_ = 5.12, *p* = 0.0202, power (1 − β) = 0.815) conditions (Figure 1A,B). A similar pattern was evident in the two-dimensional vertical plane, with significant effects in both acute (F_(2, 15)_ = 4.53, *p* = 0.0290, power (1 − β) = 0.763) and chronic (F_(2, 15)_ = 5.41, *p* = 0.0170, power (1 − β) = 0.837) conditions (Figure 1C,D). Likewise, significant differences were observed in the two-dimensional horizontal plane in both acute (F_(2, 15)_ = 8.59, *p* = 0.0033, power (1 − β) = 0.964) and chronic (F_(2, 15)_ = 8.79, *p* = 0.0030, power (1 − β) = 0.967) conditions (Figure 1E,F).

No significant differences were found between 18 °C and 26 °C. However, inter-fish distance was significantly reduced at 34 °C, particularly under chronic exposure, with differences emerging in the comparisons between 34 °C and 26 °C across all spatial dimensions (Figure 1). Under acute conditions, a significant reduction at 34 °C was observed only in the horizontal plane compared to 26 °C. These findings indicate that high temperature promotes increased shoal cohesion, as reflected by reduced inter-fish distance, especially with prolonged exposure.

**Figure 2:**
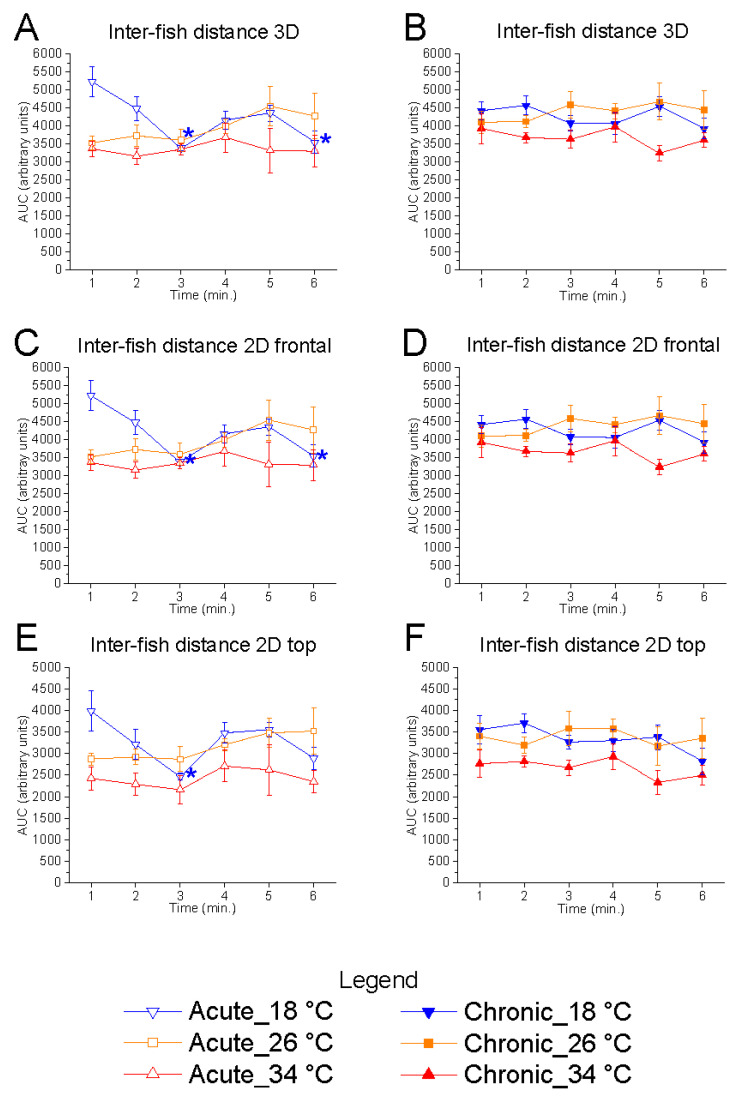
Effects of time on inter-fish distance. Effects of acute (**A**,**C**,**E**) and chronic (**B**,**D**,**F**) thermal exposure over time on inter-fish distance in 3D space (**A**,**B**), 2D vertical plane (**C**,**D**), and 2D horizontal plane (**E**,**F**). Detailed statistical values are reported in Table S1. Statistical significance: *p* ≤ 0.05. *, @, $ indicate significant differences from minute 1 at 18 °C, 26 °C, and 34 °C, respectively. Colours and geometric shapes indicate experimental temperatures: downward triangle (blue) = 18 °C; square (orange) = 26 °C; upward triangle (red) = 34 °C. Open symbols indicate acute treatment; filled symbols indicate chronic treatment.

Temporal analysis over the 6 min test period revealed no significant variation in inter-fish distance at either 26 °C or 34 °C (Figure 2). However, under acute exposure to 18 °C, a significant effect of time was detected in 3D (F_(5, 25)_ = 5.81, *p* = 0.0011, power (1 − β) = 0.988), 2D vertical (F_(5, 25)_ = 5.98, *p* = 0.0009, power (1 − β) = 0.989), and 2D horizontal planes (F_(5, 25)_ = 3.98, *p* = 0.0086, power (1 − β) = 0.900), characterised by higher inter-fish distances at the start of the test followed by a decline. No such effect was observed at 18 °C under chronic conditions. These results suggest that acute exposure to low temperatures induces a dynamic modulation of inter-fish distance, a response absent at 26 °C and 34 °C.

#### 3.1.2. Shoal Dimension Analysis

**Figure 3:**
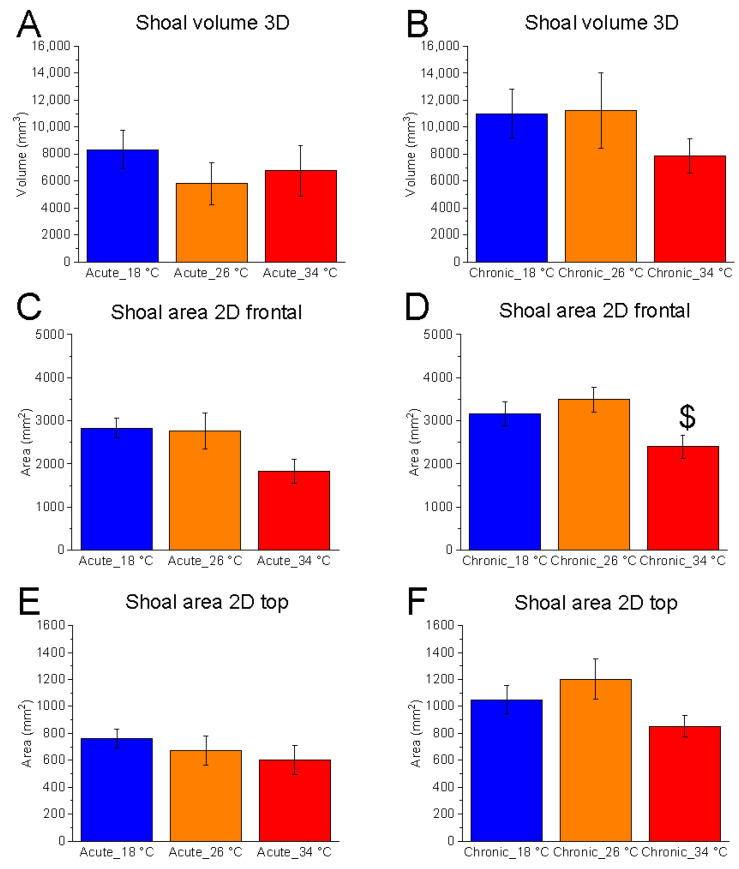
Effects of temperature on shoal dimensions. Effects of acute (**A**,**C**,**E**) and chronic (**B**,**D**,**F**) thermal exposure on shoal volume in 3D space (**A**,**B**), shoal area in the 2D vertical plane (**C**,**D**), and shoal area in the 2D horizontal plane (**E**,**F**). Detailed statistical values are reported in Table S1. Statistical significance: *p* ≤ 0.05. * 18 °C vs. 26 °C; # 18 °C vs. 34 °C; $ 26 °C vs. 34 °C. Colours: blue = 18 °C, orange = 26 °C, red = 34 °C.

Shoal dimensions, measured as 3D volume and 2D area in both vertical and horizontal planes, showed no significant temperature effect in most conditions. The exception was the vertical-plane area under chronic exposure (F_(2, 15)_ = 4.07, *p* = 0.0386, power (1 − β) = 0.715) (Figure 3A–F).

No significant differences were found between 18 °C and 26 °C. However, chronic exposure to 34 °C resulted in a reduction in vertical shoal area, suggesting increased shoal cohesion in the vertical plane (Figure 3D).

**Figure 4:**
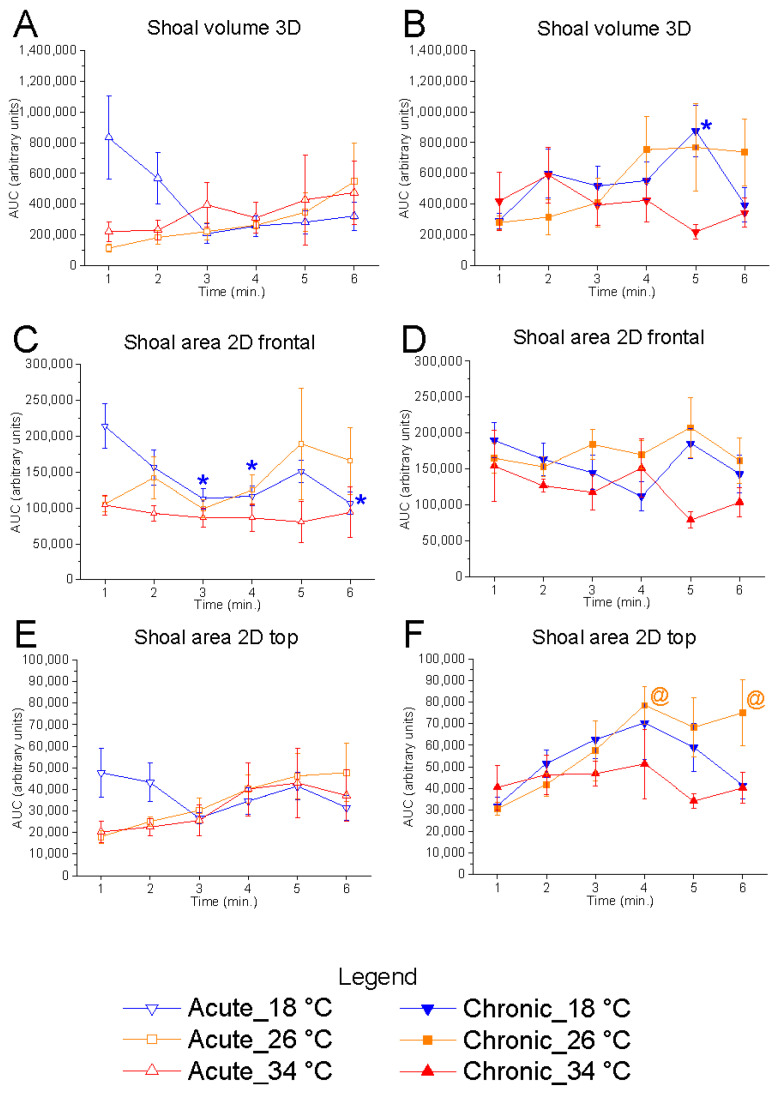
Effects of time on shoal dimensions. Effects of acute (**A**,**C**,**E**) and chronic (**B**,**D**,**F**) thermal exposure over time on shoal volume in 3D space (**A**,**B**), shoal area in the 2D vertical plane (**C**,**D**), and shoal area in the 2D horizontal plane (**E**,**F**). Detailed statistical values are reported in Table S1. Statistical significance: *p* ≤ 0.05. *, @, $ indicate significant differences from minute 1 at 18 °C, 26 °C, and 34 °C, respectively. Colours and geometric shapes: downward triangle (blue) = 18 °C; square (orange) = 26 °C; upward triangle (red) = 34 °C. Open symbols = acute treatment; filled symbols = chronic treatment.

Temporal analysis, performed using one-repeated-measures ANOVA followed by Bonferroni’s post hoc test, revealed a significant effect of time on 2D shoal area (frontal view) at 18 °C under acute conditions (F_(5, 25)_ = 4.68, *p* = 0.0037, power (1 − β) = 0.919), characterised by a progressive reduction in values over the course of the test compared to the first minute—indicative of increased vertical shoaling as the test progressed (Figure 4). Conversely, no effect of time was observed at 26 °C and 34 °C. Interestingly, a tendency to increase shoal area during the test was observed at the control temperature but not at 18 °C and 34 °C.

#### 3.1.3. Shoal Cohesion

**Figure 5:**
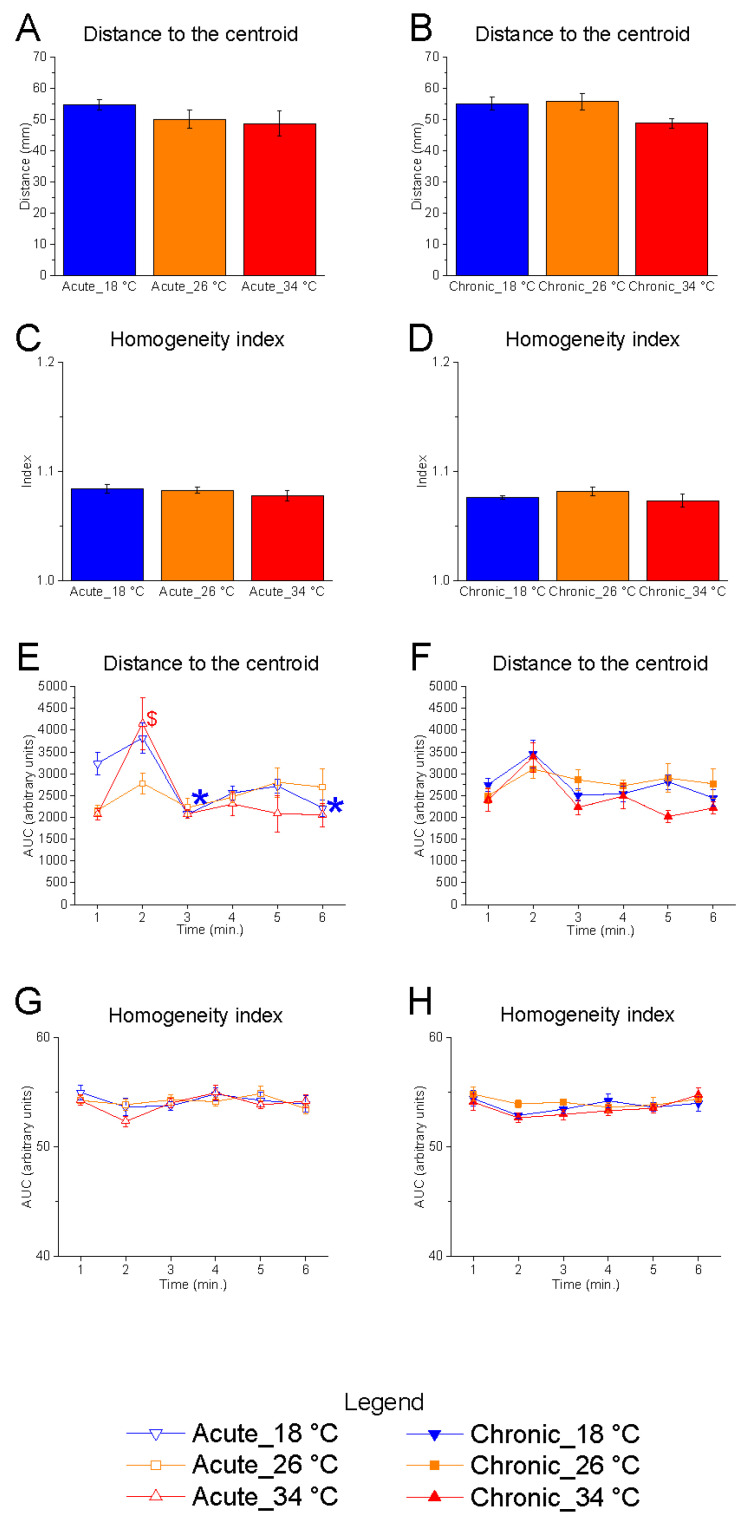
Effects of temperature and time on distance to the centroid and homogeneity index. Effects of acute (**A**,**C**,**E**,**G**) and chronic (**B**,**D**,**F**,**H**) thermal exposure on distance to the centroid (**A**,**B**,**E**,**F**) and homogeneity index (**C**,**D**,**G**,**H**). Detailed statistical values are reported in Table S1. Statistical significance: *p* ≤ 0.05. (**A**–**D**) * 18 °C vs. 26 °C; # 18 °C vs. 34 °C; $ 26 °C vs. 34 °C. (**E**–**H**) *, @, $ indicate significant differences from minute 1 at 18 °C, 26 °C, and 34 °C, respectively. Colour and symbol legend: downward triangle (blue) = 18 °C; square (orange) = 26 °C; upward triangle (red) = 34 °C. Open symbols = acute treatment; filled symbols = chronic treatment.

Neither the distance to the centroid nor the homogeneity index was significantly affected by temperature under either acute or chronic exposure conditions (Figure 5A–D).

Temporal analysis revealed no significant effect of time on either parameter at the control temperature (26 °C) (Figure 5E–H). In contrast, a progressive reduction in distance to the centroid was observed under acute exposure to 18 °C, suggesting a gradual increase in shoal cohesion during the course of the test (Figure 5E). This modulation was not observed under chronic exposure to 18 °C, indicating a possible adaptive stabilisation over time (Figure 5F).

At 34 °C, under acute conditions, a transient modulation of the distance to the centroid was noted: specifically, an increase in distance was observed at the second minute compared to the first, potentially reflecting a brief disruption in shoal cohesion during the initial phase of the test (Figure 5E).

### 3.2. Shoal Positioning

#### 3.2.1. Shoal Position on the Vertical Plane

**Figure 6:**
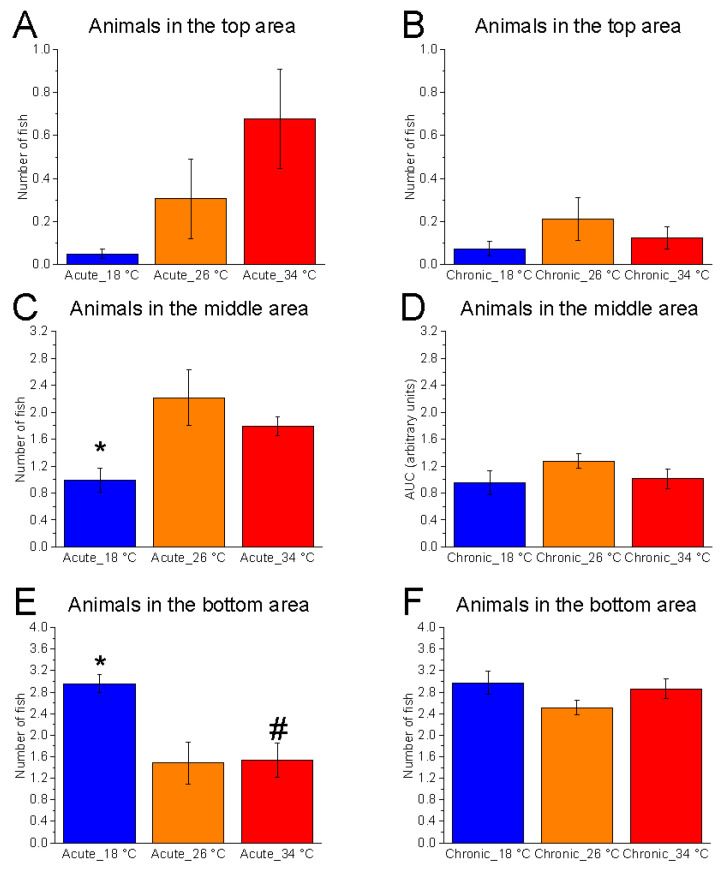
Effects of temperature on vertical shoal distribution in the vertical plane. Effects of acute (**A**,**C**,**E**) and chronic (**B**,**D**,**F**) thermal exposure on the number of zebrafish present in the top (**A**,**B**), middle (**C**,**D**), and bottom (**E**,**F**) tank zones. Detailed statistical values are reported in Table S1. Statistical significance: *p* ≤ 0.05. * 18 °C vs. 26 °C; # 18 °C vs. 34 °C; $ 26 °C vs. 34 °C. Colours: blue = 18 °C, orange = 26 °C, red = 34 °C.

Vertical distribution within the tank was significantly affected by temperature under acute conditions. Significant effects were observed in the bottom (F_(2, 15)_ = 7.34, *p* = 0.0060, power (1 − β) = 0.933) and middle (F_(2, 15)_ = 5.22, *p* = 0.0190, power (1 − β) = 0.823) zones (Figure 6). At 18 °C, there was a marked increase in the number of fish in the bottom area and a corresponding reduction in the middle zone compared to the control temperature, suggesting that acute exposure to low temperatures alters shoal positioning in the vertical plane. Conversely, no significant effects were observed at 34 °C relative to the control. Chronic exposure did not result in significant changes at either 18 °C or 34 °C, suggesting that prolonged exposure may elicit adaptive responses in the zebrafish shoal.

**Figure 7:**
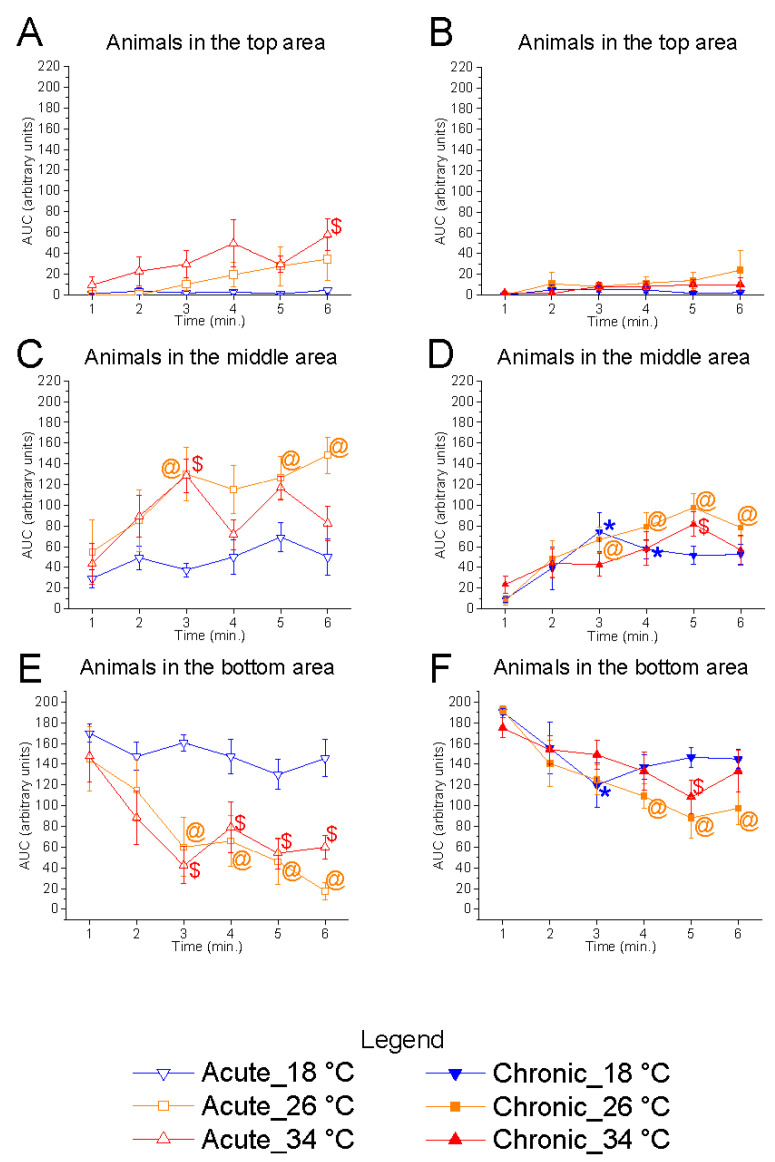
Effects of time on vertical shoal distribution in the vertical plane. Effects of acute (**A**,**C**,**E**) and chronic (**B**,**D**,**F**) thermal exposure over time on the number of zebrafish in the top (**A**,**B**), middle (**C**,**D**), and bottom (**E**,**F**) tank zones. Detailed statistical values are reported in Table S1. Statistical significance: *p* ≤ 0.05. *, @, $ indicate significant differences from minute 1 at 18 °C, 26 °C, and 34 °C, respectively. Colours and symbols: downward triangle (blue) = 18 °C; square (orange) = 26 °C; upward triangle (red) = 34 °C. Open symbols = acute treatment; filled symbols = chronic treatment.

Temporal analysis of bottom-zone occupancy showed a significant effect of time at 26 °C in both acute (F_(5, 25)_ = 7.88, *p* < 0.0001, power (1 − β) = 1.000) and chronic (F_(5, 25)_ = 5.80, *p* = 0.0011, power (1 − β) = 0.915) conditions, characterised by a gradual migration of fish from the bottom towards the upper zones (Figure 7). A similar trend was observed at 34 °C, both in acute (F_(5, 25)_ = 7.81, *p* = 0.0002, power (1 − β) = 1.000) and chronic (F_(5, 25)_ = 2.64, *p* = 0.0458, power (1 − β) = 0.809) exposure. In contrast, no significant temporal variation was observed at 18 °C under acute conditions (F_(5, 25)_ = 1.38, *p* = 0.2637, power (1 − β) = 0.542), confirming a strong preference of the shoal for the bottom area. However, under chronic 18 °C exposure, a significant effect of time was detected (F_(5, 25)_ = 3.70, *p* = 0.0122, power (1 − β) = 0.953), indicating some degree of behavioural modulation over time.

These results suggest that low temperature impairs natural exploratory behaviours during acute exposure, limiting zebrafish to the bottom zone, while chronic exposure may partially restore typical movement patterns. The higher statistical power observed in the temporal analyses reinforces the reliability of these effects over time, supporting the hypothesis that behavioural changes are dynamic and evolve throughout the trial.

#### 3.2.2. Shoal Position on the Horizontal Plane

**Figure 8:**
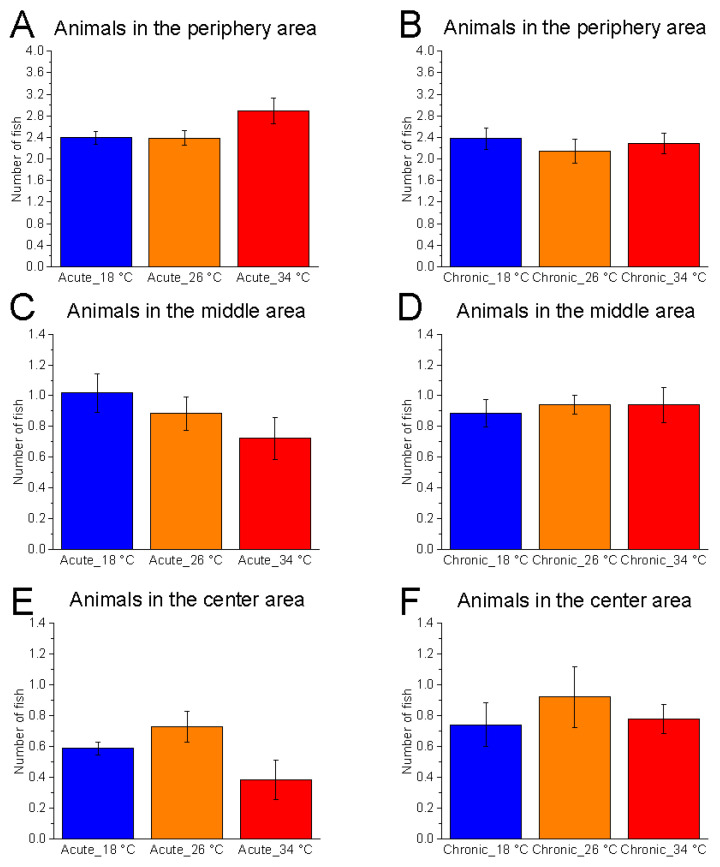
Effects of temperature on horizontal shoal distribution in the horizontal plane. Effects of acute (**A**,**C**,**E**) and chronic (**B**,**D**,**F**) thermal exposure over time on the number of zebrafish in the periphery (**A**,**B**), middle (**C**,**D**), and centre (**E**,**F**) zones. Detailed statistical values are reported in Table S1. Statistical significance: *p* ≤ 0.05. * 18 °C vs. 26 °C; # 18 °C vs. 34 °C; $ 26 °C vs. 34 °C. Colours: blue = 18 °C, orange = 26 °C, red = 34 °C.

The horizontal distribution of zebrafish was not significantly affected by temperature under either acute (number of fish in the central area, F_(2, 15)_ = 3.16, *p* = 0.0717, power (1 − β) = 0.598) or chronic (F_(2, 15)_ = 0.40, *p* = 0.6788, power (1 − β) = 0.114) conditions (Figure 8).

**Figure 9:**
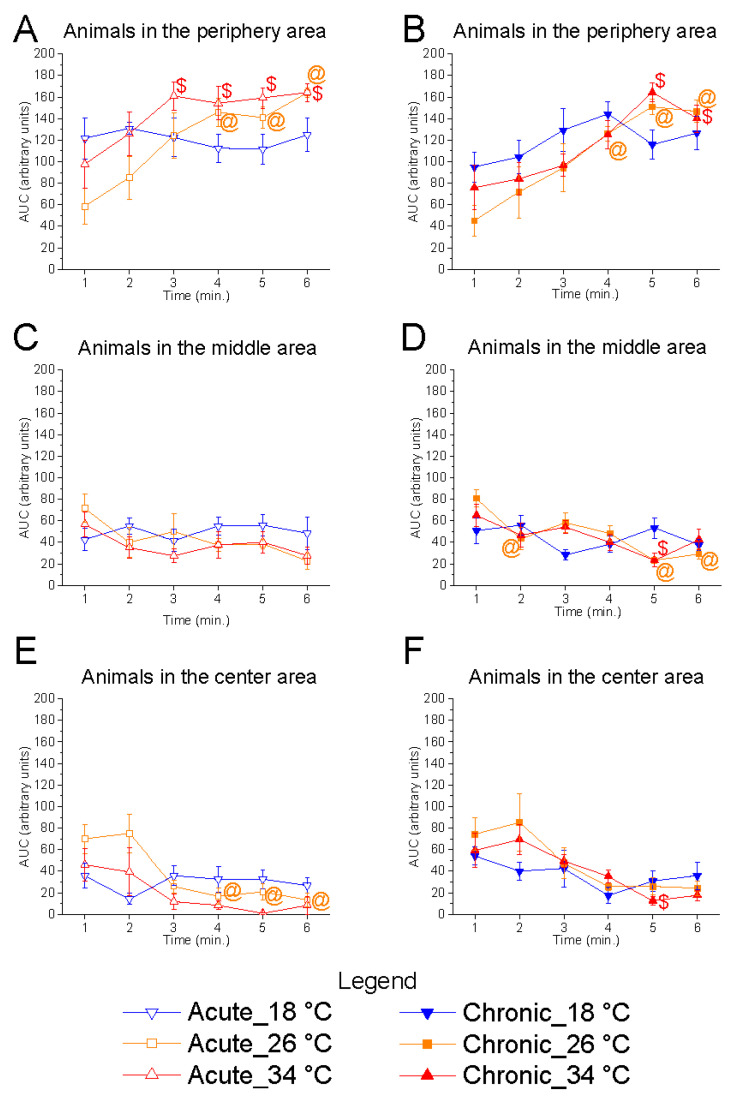
Effects of time on horizontal shoal distribution in the horizontal plane. Effects of acute (**A**,**C**,**E**) and chronic (**B**,**D**,**F**) thermal exposure over time on the number of zebrafish in the periphery (**A**,**B**), middle (**C**,**D**), and centre (**E**,**F**) zones. Detailed statistical values are reported in Table S1. Statistical significance: *p* ≤ 0.05. @, $ indicate significant differences from minute 1 at 26 °C, and 34 °C, respectively. Colours and symbols: downward triangle (blue) = 18 °C; square (orange) = 26 °C; upward triangle (red) = 34 °C. Open symbols = acute treatment; filled symbols = chronic treatment.

Temporal analysis revealed a significant effect of time at 26 °C in both acute (animals in the central area, F_(5, 25)_ = 7.44, *p* = 0.0002, power (1 − β) = 0.980) and chronic (animals in the central area, F_(5, 25)_ = 4.93, *p* = 0.0028, power (1 − β) = 0.899) conditions, characterised by a progressive reduction in the number of fish occupying the central area and an increase in the peripheral area (Figure 9). Similar trends were observed at 34 °C under both acute (F_(5, 25)_ = 3.17, *p* = 0.0238, power (1 − β) = 0.615) and chronic (F_(5, 25)_ = 6.18, *p* = 0.0007, power (1 − β) = 0.969) exposure. In contrast, no significant effect of time was observed at 18 °C in either condition, suggesting that at low temperature, the shoal did not modulate its exploration of the horizontal plane during the test.

## 4. Discussion

This study investigated the impact of thermal variation on shoaling behaviour in adult zebrafish (*Danio rerio*) by evaluating the influence of low (18 °C) and high (34 °C) temperatures—administered for either 4 days (acute exposure) or 21 days (chronic exposure)—on shoals composed of four individuals, relative to a control temperature of 26 °C. Two fundamental dimensions of shoaling behaviour were assessed: shoal structure (including inter-fish distance, volume, and area) and shoal spatial positioning (both vertical and horizontal).

Previous literature suggests that these behavioural features are sensitive to pharmacological modulation and may reflect emotional states. Specifically, exposure to anxiogenic or anxiolytic agents has been shown to alter inter-fish distance and spatial positioning, thereby providing a behavioural proxy for anxiety or boldness [17,35,37].

### 4.1. Effects of Low Temperature (18 °C)

Both acute and chronic exposure to 18 °C did not significantly affect shoal dimensions compared to the control temperature (26 °C). However, under acute exposure, a progressive reduction in inter-fish distance was observed during the 6 min test. This temporal trend—absent under control conditions—may indicate a growing anxiety-like state, consistent with previous studies in which increased shoal cohesion (i.e., reduced inter-fish distance) was induced by anxiogenic stimuli such as caffeine or conspecific alarm substances [35,37]. Conversely, decreased shoal cohesion has been reported following the administration of anxiolytics, including diazepam, alcohol, and nicotine [38,39,40,41].

Additionally, fish acutely exposed to 18 °C exhibited a significant preference for the bottom zone, with no notable changes in horizontal distribution. This vertical preference was sustained over time and contrasted with the control group, which progressively shifted towards the upper zones. Such behaviour is consistent with anxiety-related responses reported in zebrafish exposed to anxiogenic compounds [35]. Under natural conditions, this behavioural pattern represents a classic anti-predatory response, particularly against aerial predators.

These group-level behavioural patterns are consistent with findings from individual-based assays under the same thermal conditions, such as the Novel Tank Test (NTT) and the Light/Dark Test (LDT), in which fish spent more time in the bottom or dark zones—both indicative of heightened anxiety [13,14,33].

Notably, the absence of differences in average inter-fish distance between 18 °C and 26 °C suggests that social buffering may attenuate the expression of anxiety-related behaviour in groups compared to individuals.

Interestingly, the anxiety-related behaviours observed during acute exposure were no longer evident after 21 days, suggesting the activation of adaptive mechanisms over time that modulate the behavioural response to cold stress. It is important to note that zebrafish tested under chronic conditions had previously experienced the testing tank during the acute phase. Although no direct statistical comparisons were made between acute and chronic treatments, this environmental familiarity may have influenced certain behavioural outcomes. In particular, the attenuation of anxiety-like vertical positioning observed after 21 days could partially reflect a reduced novelty-induced anxiety response. This possibility is consistent with existing literature showing that prior exposure to novel environments can diminish anxiety-related behaviours in zebrafish [42]. While our study design focused on within-condition comparisons, we acknowledge that the prior experience of the testing context represents a potential confounding factor that should be considered when interpreting behavioural adaptation over time.

### 4.2. Effects of High Temperature (34 °C)

Exposure to 34 °C resulted in a significant increase in shoal cohesion, as evidenced by a reduction in inter-fish distance compared to the control. This effect was already detectable during acute exposure (particularly in the horizontal plane) and became more pronounced under chronic conditions, with consistent reductions across all measured dimensions (3D, vertical, and horizontal). These results indicate that 21 days of exposure did not elicit behavioural compensation; on the contrary, deviations from control behaviour became more marked. Such anxiety-related responses appear to be robustly embedded as an innate or stereotyped social cohesion response.

Unlike under low-temperature conditions, no intra-test modulation was observed, and shoal cohesion remained high throughout the test.

Although increased shoaling is often interpreted as an anxiety-like response, this finding contrasts with individual-based assays in which zebrafish exposed to 34 °C displayed enhanced exploration of the upper (NTT) and light (LDT) zones—both typically associated with boldness and reduced anxiety [13,14,33].

Our analysis of shoal positioning revealed no significant differences in vertical or horizontal exploration between 34 °C and 26 °C. However, temporal analysis during acute exposure revealed a progressive reduction in bottom zone occupancy and a current increase in the use of periphery, suggesting active environmental exploration.

This apparent discrepancy between individual and group-level responses underscores the importance of social context in behavioural assessments. While individual fish may display boldness under thermal stress, groups appear to adopt more cohesive behaviours—potentially as coping or thermoregulatory strategies. However, in the absence of physiological indicators such as cortisol levels or metabolic rate data, such interpretations remain speculative. While the observed increase in shoal cohesion may reflect a coping or thermoregulatory response, further studies incorporating endocrine and metabolic parameters are required to confirm the underlying mechanisms.

Shoaling confers multiple benefits, including predator avoidance, enhanced foraging efficiency, and energy conservation via hydrodynamic effects [43]. The increase in shoal cohesion at 34 °C may thus serve both as an adaptive behavioural response and as a thermoregulatory strategy that facilitates the collective exploration of marginal environments. However, such benefits are constrained in laboratory settings, where spatial limitations and uniform temperature conditions prevent relocation to optimal microhabitats.

The persistence of increases in shoaling behaviour following 21 days of exposure at 34 °C suggests that this duration is insufficient to trigger behavioural adaptation. Supporting this hypothesis, Mukherjee and Bhat [44] reported no significant differences in shoaling behaviour after 45 days of exposure to 31 °C, suggesting that longer exposures may be necessary to activate homeostatic mechanisms that restore behavioural normalcy.

### 4.3. Proteomic and Behavioural Correlations

Previous proteomic and lipidomic studies have shown that exposure to both 18 °C and 34 °C induces significant changes in the brains of adult zebrafish. Specifically, thermal stress altered the expression of proteins involved in critical neurophysiological processes—most notably, those linked to synaptic vesicle transport—and disrupted lipid homeostasis [14,32,33,34].

These molecular changes are likely to affect neural communication and may underlie the observed behavioural phenotypes. Behavioural assays conducted under identical conditions (NTT, LDT, social preference test, mirror biting test, and Y-maze) have demonstrated that both 18 °C and 34 °C produce distinct neurobehavioural profiles in individual zebrafish—low temperatures inducing anxiety-like traits and high temperatures being associated with boldness [13,14,34].

The present study expands on these findings by demonstrating that temperature-induced behavioural alterations are also evident at the group level in small shoals, thereby reinforcing the link between neurochemical changes and collective behaviour. Notably, at 18 °C, shoaling behaviour aligned with an anxiety-like profile, whereas behavioural outcomes at 34 °C were more complex—characterised by increased cohesion without a clear expression of either anxiety or boldness. This suggests that social interactions may modulate or buffer thermally induced behavioural changes in nuanced ways.

Further research is needed to elucidate the neurobiological mechanisms driving these responses and to determine whether the behavioural alterations observed under thermal stress represent maladaptive outcomes or adaptive strategies. Nonetheless, it is important to acknowledge that the sample size—comprising six shoals per temperature group—was relatively limited, which may constrain the broader generalisability of the findings. However, to address this potential limitation, we performed a post hoc power analysis (1 − β), which indicated that the statistical tests employed had adequate power to detect the observed effects. This supports the robustness of the behavioural outcomes reported, despite the modest level of replication. Future studies involving larger sample sizes would nonetheless be valuable to validate these findings and further explore variability across shoals.

## 5. Conclusions

Environmental temperature variation influences the spatio-temporal shoaling behaviour of adult zebrafish (*Danio rerio*) in a manner dependent on both temperature and duration of exposure. The findings indicate that (1) exposure to elevated temperatures (34 °C) enhances shoal cohesion; (2) these behavioural modifications become more pronounced following prolonged exposure, supporting the notion of a cumulative effect of thermal stress over time; (3) exposure to low temperatures (18 °C) alters the shoal’s exploratory pattern, notably increasing the occupancy of the lower portion of the tank during the acute phase of exposure—an effect consistent with anxiety-like responses; and (4) such behavioural changes are likely driven by temperature-induced shifts in brain neurochemistry and individual behavioural states, which in turn modulate the emergent properties of collective behaviour.

Taken together, these results underscore the sensitivity of social behaviours in ectothermic species to environmental thermal fluctuations and highlight the ecological significance of temperature in shaping group-level dynamics.

## Data Availability

The raw data supporting the conclusions of this article will be made available by the authors on request.

## Supplementary Materials

The following supporting information can be downloaded at https://www.mdpi.com/article/10.3390/ani15142006/s1: Table S1: Summary of statistical results (F, *p*, and statistical power [1 − β]) for the behavioural parameters presented in Figure 1, Figure 2, Figure 3, Figure 4, Figure 5, Figure 6, Figure 7, Figure 8 and Figure 9.

## Abbreviations

The following abbreviations are used in this manuscript:

2D	Two-dimensional
3D	Three-dimensional
ANOVA	Analysis of Variance
AUC	Area under the curve
LDT	Light/Dark Test
NTT	Novel Tank Test
S.E.M.	standard error of the mean
